# Nonlinear Optimization of Orthotropic Steel Deck System Based on Response Surface Methodology

**DOI:** 10.34133/2020/1303672

**Published:** 2020-04-21

**Authors:** Wei Huang, Minshan Pei, Xiaodong Liu, Chuang Yan, Ya Wei

**Affiliations:** ^1^Intelligent Transportation System Research Center, Southeast University, Nanjing 210096, China; ^2^CCCC Highway Consultants Co., Ltd., Beijing 100084, China; ^3^Department of Civil Engineering, Tsinghua University, Beijing 100084, China

## Abstract

The steel bridge deck system, directly subjected to the vehicle load, is an important component to be considered in the optimization design of the bridges. Due to its complex structure, the design parameters are coupled with each other, and many fatigue details in the system result in time-consuming calculation during structure optimization. In view of this, a nonlinear optimization method based on the response surface methodology (RSM) is proposed in this study to simplify the design process and to reduce the amount of calculations during optimization. The optimization design of the steel bridge deck system with two-layer pavement on the top of the steel deck plate is taken as an example, the influence of eight structural parameters is considered. The Box-Behnken design is used to construct a sample space in which the eight structural parameters can be distributed evenly to reduce the calculation workload. The finite element method is used to model the mechanical responses of the steel bridge deck system. From the regression analysis by the RSM, the explicit relationships between the fatigue details and the design parameters can be obtained, based on which the nonlinear optimization design of the bridge deck system is conducted. The influence of constraint functions, objective functions, and optimization algorithms is also analyzed. The method proposed in this study is capable of considering the influence of different structural parameters and different optimization objectives according to the actual needs, which will effectively simplify the optimization design of the steel bridge deck system.

## 1. Introduction

China has constructed hundreds of long-span steel bridges since the 1990s in the last century and accumulated a lot of experience in the design and construction of such bridges. Orthotropic steel box girder is the main structural form of stiffening girders for long-span bridges at present. It has the advantages of light weight, strong ultimate bearing capacity, easy assembly, and construction. However, the related design methods are still inadequate, and the fatigue failure of orthotropic steel bridge deck system is prominent and has not been effectively solved in recent years. It is necessary to investigate the optimization design method of orthotropic steel decks for long-span bridges to improve their safety and economy.

The steel bridge deck system mainly includes the orthotropic steel plate and the pavement on the top of the plate, which directly bears the repeated traffic loads. Due to the complex structure and the characteristics of orthotropic, it is difficult to use the analytical method to guide the optimization design of the steel bridge deck system. Instead, the finite element method is generally used to carry out the related optimization design.

In recent years, the optimization methods of the steel bridge deck system have developed from the single-parameter method to the multiparameter method. In the single-parameter method [1], only the value of a single parameter of the structure is varied during the optimization process, and other parameters are kept constant. Based on a large number of calculations, the strength and stiffness of the structure can be obtained to determine the structural parameters of the bridge deck system. The single-parameter method can neither take into account the coupling effects from different structural parameters of the bridge deck system nor provide the best design solution. Yu [2] and Zhao and Qian [3] used the optimization design module of the commercial finite element software to carry out the structural design of the bridge deck system and determined the best design solution that met the safety requirements through multiple iterative calculations. This solution can take into account multiple structural parameters. However, there are problems such as large amount of calculations and analysis. Zhuang and Miao [4] proposed the optimization method by utilizing the combination of neural network and genetic algorithm with the objective of improving welding performance of the orthotropic steel bridge deck and established the relationship between the structural parameters and the equivalent stress amplitude to guide the optimization design of the orthotropic steel bridge deck system. This method can take into account the effects of multiple structural parameters. However, the neural network training is complicated, and the model ability to accurately predict the results of untrained samples remains to be further verified.

The response surface methodology (RSM) is an effective way to solve the multiparameter optimization problem. The response values are obtained by experiments, and RSM uses multiple regression equations to establish the relationships between the structural parameters and the response values. The optimized structural parameters are finally determined according to the optimization objective. RSM has the advantages of simplifying calculation and predicting the result of the randomly combined parameters. Since being proposed, RSM has been widely used to solve the optimization problems in fields of microorganisms [5], food [6], petrochemical [7], environmental protection [8], chemistry [9], etc. Some researchers have used RSM to optimize the design of steel bridge decks. Ma [10] analyzed the stress response of the weak parts of orthotropic steel bridge deck based on finite element modeling. He used RSM to establish the response surface model to analyze the stress of various critical parts and optimized the design of the steel bridge deck system to improve each single fatigue detail (i.e., stress, strain, or deflection of a certain part of the system).

Cui et al. [11] used the multiobjective design method to conduct the optimization of the plain orthotropic steel bridge deck. However, the influence of both the pavement layer and the local stiffness of the orthotropic steel bridge deck system on its fatigue performance was not considered. Existing studies have shown that the structure of the pavement layer is an important parameter affecting the stress state of the bridge deck system by reducing the stress and deflection of the orthotropic steel plate [12]. Therefore, the influence of the pavement layer has to be considered during optimization.

Due to the mutual coupling effect from different fatigue details of the steel bridge deck system, it is necessary to carry out research on optimization design that multiple fatigue details can be considered to improve the rationality and accuracy of the optimized results. This study proposes a nonlinear optimization method for the design of the steel bridge deck system based on the response surface methodology. A finite element model is developed to analyze the mechanical response of the samples. The explicit relationships between the six fatigue details and the eight structural parameters are obtained through the response surface methodology, based on which the nonlinear optimization design of the bridge deck system is conducted. The influence of constraint functions, objective functions, and the optimization algorithms on the results of nonlinear optimization is analyzed.

Compared to previous research, this study takes into account the influence of steel orthotropic plate and pavement parameters on the structural performance of the steel bridge deck system. Because this study combines RMS and nonlinear optimization, different objectives can be quickly realized based on the objective functions and the constraint functions after the explicit functional relationships between the fatigue details and the structural parameters are obtained by RMS.

## 2. The Overall Process of Nonlinear Multiobjective Optimization

Due to the complexity of the steel bridge deck system, the finite element method is normally used to analyze the structural responses such as stress, strain, and deflection. However, the computation workload will increase significantly for optimization problems with multiple objectives, which results in a reduction in design efficiency and is unfavorable to the engineering applications. To improve this situation, this study proposes to use the response surface methodology for the nonlinear optimization design.

**Figure 1:**
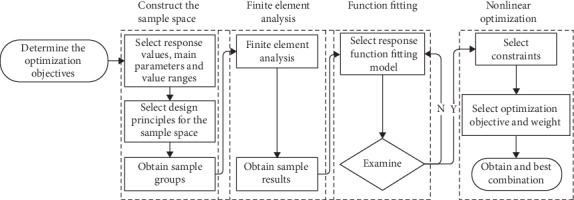
The process of nonlinear optimization based on response surface methodology.

The method mainly includes four steps: sample group construction, finite element modeling, function fitting, and nonlinear optimization, as shown in Figure 1. After determining the optimization objectives, it is necessary to select structural parameters, response values, and value ranges of the optimization design. The response values of the samples are obtained from the FE analysis, which are used for establishing the explicit relationships between the response values and the structural parameters. Based on the design requirements, the constraint conditions, the optimization objectives, and the weights of each objective are selected. The nonlinear optimization analysis is finally conducted to obtain the optimized design results.

## 3. Sample Space Construction Based on RSM

### 3.1. Fundamental Principles of Response Surface Methodology (RSM)

In view of the complex parameters of the steel bridge deck system and their coupling effects, this study utilizes the response surface methodology (RSM) to carry out the sample group construction of the steel bridge deck system. The explicit relationships between the structural parameters and the response of the steel bridge deck system are obtained from the calculated results of samples by finite element analysis. The multiple quadratic regression equations are normally used in the RSM to obtain the explicit relationships between the response values and the structural parameters, which is a common method for solving multivariable optimization problems to seek the most optimal structural parameters.

**Figure 2:**
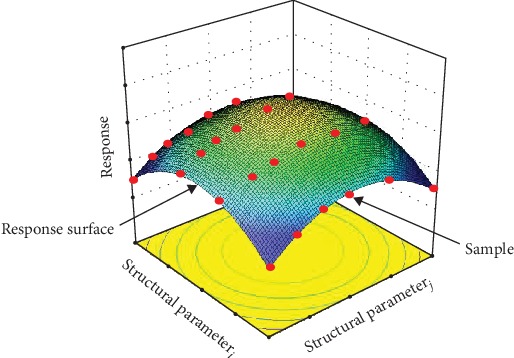
The diagram of response surface representing the explicit relationship between response value and structural parameters.

Figure 2 is a schematic diagram of the constructed response surface with two parameters by RSM. The red scattered data points on the response surface are the initial samples. To obtain an accurate relationship between the response values and the parameters, the initial samples are evenly distributed in the design space. The response surface in Figure 2 is obtained by the regression analysis based on the response values and the parameters, which generally has explicit features to facilitate the subsequent nonlinear optimization design.

### 3.2. The Main Structural Parameters and Their Value Ranges

There are many structural parameters in the steel bridge deck system, and it is challenging to consider the influence of all the structural parameters during the optimization design process. Therefore, it is necessary to firstly determine the major structural parameters and their value ranges that affect the mechanical response of the steel bridge deck system the most. In this study, the structural parameter set is expressed as follows:

(1) $$\begin{array}{c} X={\left(x_{1},x_{2},x_{3},\cdots \cdots ,x_{i},\cdots \cdots \right)}^{T}, \end{array}$$

where *x*_*i*_ is the *i*^th^ structural parameter of the steel bridge deck system.

Similarly, the response value set is expressed as follows:

(2) $$\begin{array}{c} Y={\left(y_{1},y_{2},y_{3},\cdots \cdots ,y_{j},\cdots \cdots \right)}^{T}, \end{array}$$

where *y*_*j*_ is the *j*^th^ response value.

The explicit functional relationship between the response values and the structural parameters can be expressed as follows:

(3) $$\begin{array}{c} Y={\left(f_{1}\left(X\right),f_{2}\left(X\right),f_{3}\left(X\right),\cdots \cdots ,f_{j}\left(X\right),\cdots \cdots \right)}^{T}, \end{array}$$

where *f*_*j*_(**X**) = *y*_*j*_.

The major structural parameters can be directly selected if the importance of each one is known before the optimization. If the importance of structural parameters cannot be judged in advance, the Plackett-Burman Design [13], range test [14], etc. can be used to determine the degree of influence of each structural parameter on the response values.

Existing studies have shown that for conventional steel bridge deck systems, the eight structural parameters have greater impact on the mechanical response of the system, which include the thickness of the top plate [15, 16], the thickness of the U-ribs [17], the thickness of the diaphragm [15], the spacing of the diaphragms [15], the thickness of the bottom pavement layer [17], the elastic modulus of the bottom pavement layer [18], the thickness of the top pavement layer [17], and the elastic modulus of the top pavement layer [18]. This study selects these eight structural parameters for the response surface construction.

The value ranges of the eight structural parameters are summarized based on the survey on the steel bridge deck system in China, as listed in Table 1. Other than the above eight structural parameters, other parameters are normally constant values according to the investigations on the typical large-span steel bridges in China (as shown in Table 2 [1, 19–24]). These structural parameters include the upper opening width, height, lower opening width, center distance of the U-rib, and the height of the transverse diaphragm. Correspondingly, the values of these parameters are taken as constant during the optimization. Their specific values are summarized in Table 1 as well. On the other hand, to prevent cracks in the diaphragm plate at the arc-shaped opening, AASHTO [25], Japanese Road Code [26], and Eurocode 3 [27] all provide corresponding structural parameters for the U-rib and the diaphragm plate opening. This study adopts the arc notch form of the diaphragm according to the Eurocode 3 [27].

### 3.3. Fatigue Details

**Figure 3:**
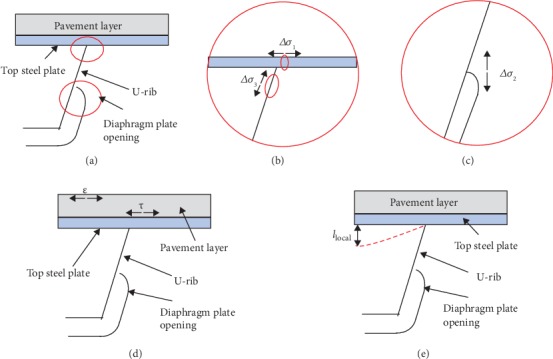
The locations of the six fatigue details. (a) Joints of orthotropic steel plate members. (b) The locations of Δ*σ*_1_ and Δ*σ*_3_. (c) The location of Δ*σ*_2_. (d) The location of *τ* and *ε*. (e) The location of *l*_local_.

Existing research shows that there are multiple fatigue details in the orthotropic steel bridge deck system, which are critical factors controlling the defects of the system [28–30]. By referring to the frequent distresses found during the bridge inspection conducted by the authors in the year of 2018 and the fatigue details specified in the Chinese code [31] as well as the relevant literatures [28–30], this study will consider the response values of six fatigue details, including the stress amplitude at the welding joint between the top plate and the U-rib in the transverse direction (Δ*σ*_1_, MPa), the stress amplitude at the opening of the diaphragm plate in the height direction (Δ*σ*_2_, MPa), the stress amplitude at the inner side of stiffener in the oblique rib direction (Δ*σ*_3_, MPa), the shear stress at the bottom pavement layer in the transverse direction (*τ*, MPa), the tensile strain at the top pavement layer in the transverse direction (*ε*), and the local deflection of the top plate (*l*_local_, mm). The positions of the six fatigue details are shown in Figure 3. The response values of the six fatigue details will be calculated numerically by the FE analysis, which are used for establishing the explicit relationships between the structural parameters and the responses for the further nonlinear optimization design.

### 3.4. Selection of Sample Design Methods

In the process of response surface construction, the design of the sample group will directly affect the accuracy of the explicit relationships to be established and further affect the results of the optimization design. The number of the samples should be neither too small nor too large. A small number of samples is not able to establish the explicit relationships to accurately represent the response in the design space. A large number of samples will significantly increase the optimization workload. In addition, the samples should be evenly distributed within the value ranges to improve the accuracy of the response surface functions which can explicitly describe the relationships between the response values and structural parameters. Therefore, the key to sample design is to determine a suitable number of samples that are evenly distributed in the design space.

At present, the commonly used sample design methods in RSM include the factorial experimental design, central composite design (referred to as “CCD”), Box-Behnken design (referred to as “BBD”), D-optimization design, and Latin square design [32–35]. Among them, the CCD method and the BBD method select samples to ensure the spatial uniform sample distribution. The uniform distribution of samples is critical for obtaining accurate explicit functions and avoiding large errors in the spaces with sparse samples. Considering that the BBD method uses fewer experiments to obtain a uniformly distributed sample group compared to the CCD method, the BBD method will be used for sample design in this study.

**Figure 4:**
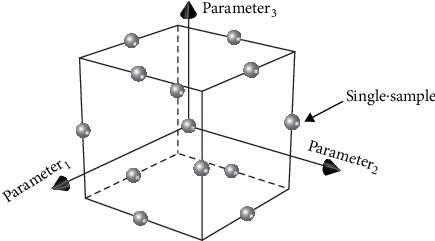
The samples in the three-parameter distribution model designed by the Box-Behnken design.

The BBD design method selects the combination of parameters at the mid-points of the edges and the center of the sample space as samples. Each parameter always has 3 levels, that is, the maximum, the minimum, and the median in the value ranges.  Figure 4 is a schematic diagram of the sample space designed by the three parameters by using the BBD method. The sample space is cubic. The dots in Figure 4 represent a group of samples which are taken at the mid-points of the edges and the center of the cube.

### 3.5. Generation of Sample Groups

According to the value range of the major structural parameters summarized in Table 1, the maximum, the minimum, and the median values of the eight structural parameters were determined. Particularly, by referring to a multidimensional space formed by the value range of the eight structural parameters, the center of the multidimensional space and the mid-point of its edge line are taken as samples. The generated sample group including a total of 120 samples is listed in Table 3.

Based on the above generated sample group, finite element analysis is conducted to calculate the mechanical responses in terms of the six fatigue details (Δ*σ*_1_, Δ*σ*_2_, Δ*σ*_3_, *τ*, *ε*, and *l*_local_) for each sample. The process of FE analysis is detailed in Section 4.

## 4. Finite Element Modeling Mechanical Responses of Steel Bridge Deck System

### 4.1. Finite Element Model

**Figure 5:**
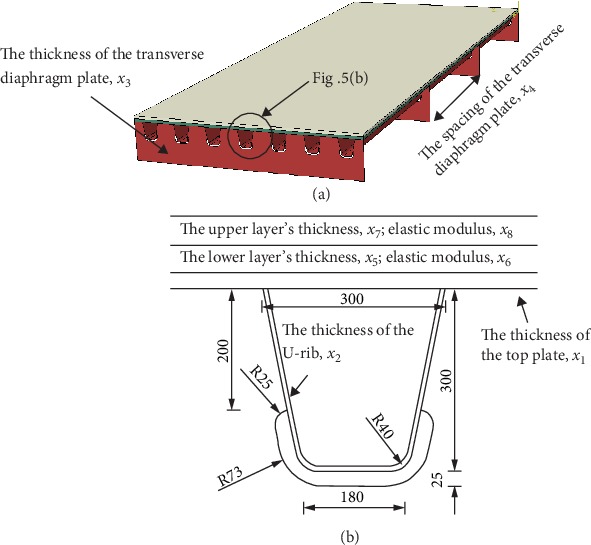
(a) FE model of bridge deck system. (b) The opening type of the transverse diaphragm plate (unit : mm).

To obtain the mechanical responses of the steel bridge deck system under the traffic loads, a finite element model is established by using the ABAQUS software, as shown in Figure 5(a). The finite element model simulates the second system of the steel box girder bridge, including the steel orthotropic plate and the pavement layer. The orthotropic steel plate is supported on the box girder, which mainly includes components such as transverse diaphragm, U-shaped stiffeners, and roof plates. The opening of the diaphragm adopts the form recommended by the Eurocode 3 [27]. The shape and the corresponding size of the opening are shown in Figure 5(b).

The finite element model of the second system of the steel box girder bridge established in this study is composed of four transverse diaphragms in the longitudinal direction and seven U-shaped stiffeners in the transverse direction. Existing research shows that this type of model can better reflect the mechanical responses of the steel bridge deck system [36, 37]. Considering the popular use of double-sided welding technology in China, the defects of steel bridge due to welding has been significantly improved. Therefore, the effects of welding defects are not considered in the modeling in this study.

The steel orthotropic plate was meshed with S4 and S3 shell elements, and the pavement layer was meshed with C3D8 solid elements. The mesh size in this study is set as 10 mm. According to the results of the trial calculation, the mesh size can reduce the calculation workload and maintain the accuracy of the calculation results. The calculation is simulated by a finite element with static implicit scheme.

### 4.2. Material Parameters

The finite element model established in this study requires the material mechanical parameters as inputs. The steel parameters are selected according to the provisions of the “Specifications for Design of Highway Steel Bridge (JTG D64-2015)” [31]. The elastic modulus, the shear modulus, the Poisson ratio, and the density of the steel are 2.06 × 10^5^ MPa, 0.79 × 10^5^ MPa, 0.31, and 7850 kg/m^3^, respectively. The effect of material defect and the impact from environment and traffic loads on the physical properties of the steel are not considered. According to the survey of the existing long-span bridges in China [19], the elastic modulus of the pavement materials ranges from 4000 to 17000 MPa, and the Poisson ratio is 0.35. The influence of temperature on the mechanical properties of pavement materials is not considered.

### 4.3. Boundary Conditions

The boundary conditions used in this study are as follows. The bottom of the diaphragms is fixed, and the two sides of the diaphragms are symmetrical about the center line in the transverse direction. There is no displacement between the top steel plate and the pavement layer in the horizontal direction. The *tie* command in ABAQUS is used to define the interface contact conditions between the pavement layer and the steel deck plate.

### 4.4. Loading Conditions

**Figure 6:**
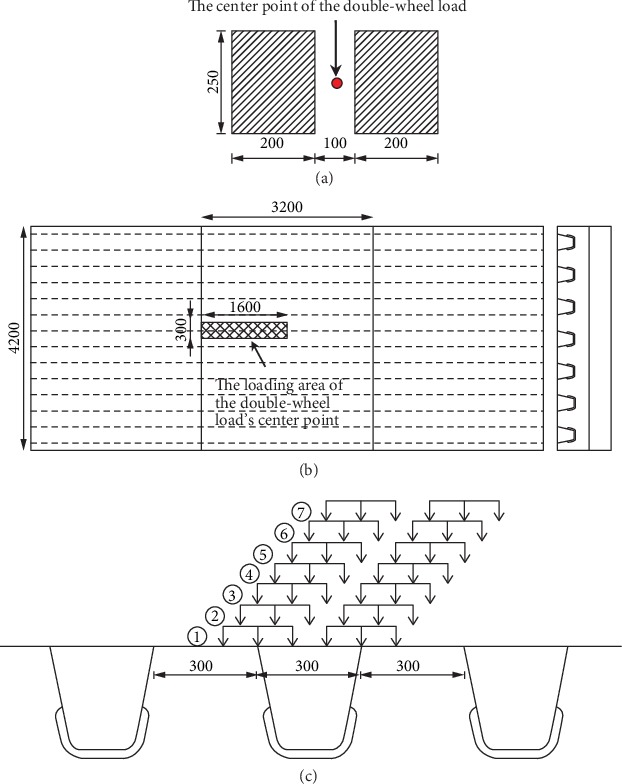
Illustration of finding the unfavorable loading position. (a) The double-wheel load model (unit : mm). (b) The load application area on the steel bridge deck system. (c) The transverse distribution of the double-wheel load.

As mentioned earlier, the mechanical response of the orthotropic steel bridge deck system has local effects. According to “Specifications for Design of Highway Steel Bridge (JTG D64-2015)” [31], a double-wheel load of 35 kN is applied in the finite element model, as shown in Figure 6(a). The area of the single wheel load is 250 mm × 200 mm, the wheel spacing is 100 mm, and the wheel pressure is 0.7 MPa.

Due to anisotropy and complex nature of the steel bridge deck structure, multiple fatigue details exist, such as Δ*σ*_1_, Δ*σ*_2_, Δ*σ*_3_, *τ*, *ε*, and *l*_local_. These fatigue details correspond different loading positions which are necessary to be identified for the critical response calculation.

The most unfavorable loading position of each fatigue detail can be identified by the trial calculation through load traversal. To reduce the calculation workload, one case with structural parameters as follows was carried out first to identify the most unfavorable loading position for each fatigue details. The thickness of the top plate is 14 mm. For U-rib, the thickness is 8 mm, the width of the upper opening is 300 mm, the width of the lower opening is 180 mm, the height is 300 mm, and the center distance between the two adjacent U-ribs is 600 mm. For the diaphragm, the thickness is 10 mm and the center distance between the two adjacent diaphragms is 3200 mm. The pavement includes two layers of epoxy asphalt mixture. The thickness of each layer is 30 mm, the elastic modulus of the pavement materials is 17000 MPa, and the Poisson ratio of the pavement materials is 0.35.

Considering the symmetry of the steel bridge deck structure, the longitudinal range of the loading area is between the second diaphragm and its mid-span, and the transverse range is between the two adjacent U-rib centerlines (Figure 6(b)). During the traversal of the double-wheel load, the longitudinal step of movement is 100 mm, and there are 17 loading positions; the transverse step of movement is 50 mm, and there are 7 loading positions (Figure 6(c)).

During the traversal of the double-wheel load, the most unfavorable loading position where the maximum stress, strain, or deflection are achieved for each fatigue detail can be determined from the finite element analysis, which will be detailed in Section 5.

## 5. Calculated Mechanical Responses by FEA

### 5.1. Maximum Transverse Stress Amplitude at Welding Joint between Top Plate and U-Rib (Δ*σ*_1_) and Its Most Unfavorable Loading Position

The fatigue cracking at the joint between the U-rib and the top plate is mainly caused by the excessive stress amplitude (Δ*σ*_1_) at the welding joint, which equals to the sum of the absolute value of the maximum tensile stress and the maximum compressive stress generated at the same position. The use of “Δ” represents that stress amplitude.

**Figure 7:**
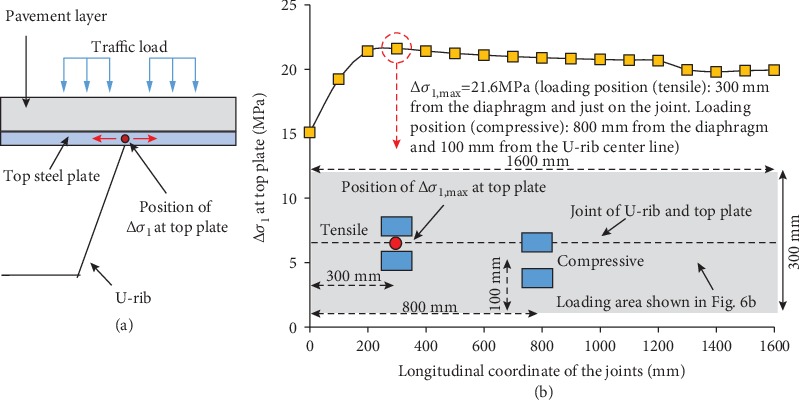
(a) The location of Δ*σ*_1_ and (b) the change of Δ*σ*_1_ along the longitudinal direction with the location of Δ*σ*_1_ and the corresponding loading positions being marked, the maximum Δ*σ*_1_, and the corresponding most unfavorable loading position are marked.

The maximum Δ*σ*_1_ can be obtained through traversal of the double-wheel load within the loading area (shown in Figure 7). It is seen that the stress amplitude varies at different locations along the joints. Δ*σ*_1_ is the smallest near the diaphragm. With the joint away from the diaphragm, Δ*σ*_1_ increases rapidly and then decreases slightly until reaching a stable state (shown in Figure 7(b)). Δ*σ*_1_ reaches the largest of 21.6 MPa at the location of 300 mm away from the diaphragm. This largest stress amplitude is generated by the summation of the maximum tensile stress and the maximum compressive stress which are caused by the load applied at 300 mm from the diaphragm and just on the joint and the load applied at 800 mm from the diaphragm and 100 mm from the U-rib center line, respectively.

### 5.2. Maximum Stress Amplitude at the Opening of Diaphragm in the Height Direction (Δ*σ*_2_) and Its Most Unfavorable Loading Position

**Figure 8:**
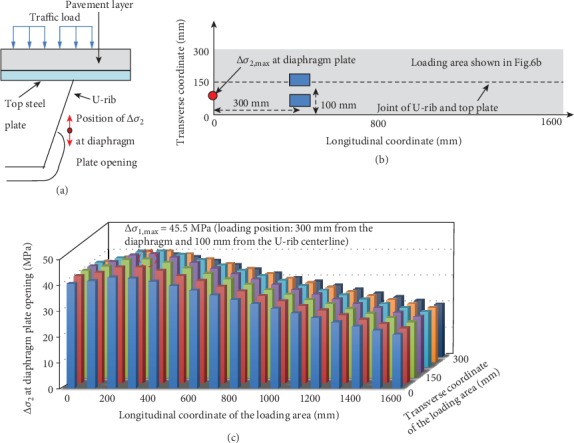
(a) The location of Δ*σ*_2_, (b) the location of Δ*σ*_2,max_ and the corresponding loading position, and (c) the change of Δ*σ*_2_ with the change of loading positions.

Similarly, the fatigue cracking at the diaphragm opening is mainly caused by the excessive stress amplitude at that location (Figure 8(a)). By load traversal through the gray area in Figure 8(b), it is found that the opening of the diaphragm is always in a tensile stress state. Therefore, the maximum stress amplitude at the opening of diaphragm in the height direction (Δ*σ*_2_) equals to the maximum tensile stress.

**Figure 9:**
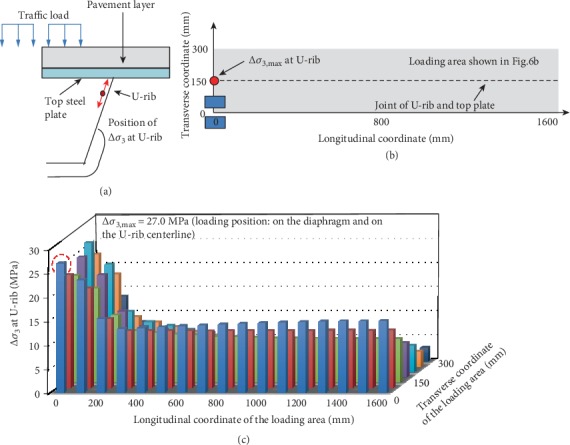
(a) The location of Δ*σ*_3_, (b) the location of Δ*σ*_3,max_ and the corresponding loading position, and (c) the change of Δ*σ*_3_ with the change of loading positions.

The relationship between Δ*σ*_2_ and the position of the double-wheel load is shown in Figure 8(c). It is seen that when the load is away from the diaphragm, Δ*σ*_2_ first increases to reach its maximum. As the double-wheel load moves further away from the diaphragm, Δ*σ*_2_ begins to decrease linearly and reaches a minimum when the double-wheel load is located at the mid-span between the two diaphragms. In particular, when the center point of the double-wheel load is 300 mm from the diaphragm and 100 mm from the U-rib centerline, Δ*σ*_2_ reaches the maximum of about 45.5 MPa. From the calculated results, Δ*σ*_2_ is larger than stress amplitudes of Δ*σ*_1_ (Figure 7) and Δ*σ*_3_ (Figure 9). Therefore, it is necessary to strengthen the thickness of the steel plate at the opening of the diaphragm or optimize the shape of the opening to prevent fatigue cracking.

### 5.3. Maximum Stress Amplitude at the Inner Side of Stiffener in the Oblique Rib Direction (Δ*σ*_3_) and Its Most Unfavorable Loading Position

Similar to the calculations of the previous two stress amplitudes, the center point of the double-wheel load is traversed and loaded in the gray area in Figure 9 to obtain the stress amplitude at the inner side of stiffener in the oblique rib direction (Δ*σ*_3_). It is found that the U-rib is always in the tensile stress state; therefore, it is considered that the stress amplitude is equal to the absolute value of the tensile stress.

The variation of Δ*σ*_3_ with different loading positions is plotted in Figure 9(c). It is seen that when the center point of the double-wheel load is located at the U-rib centerline and the transverse diaphragm, Δ*σ*_3_ is the largest of about 27.0 MPa. As the load moves further away from the diaphragm, Δ*σ*_3_ first decreases rapidly and then slight increases starting from 200 mm until stabilized.

### 5.4. Maximum Shear Stress at the Bottom of Pavement Layer (*τ*) and Its Unfavorable Loading Position

**Figure 10:**
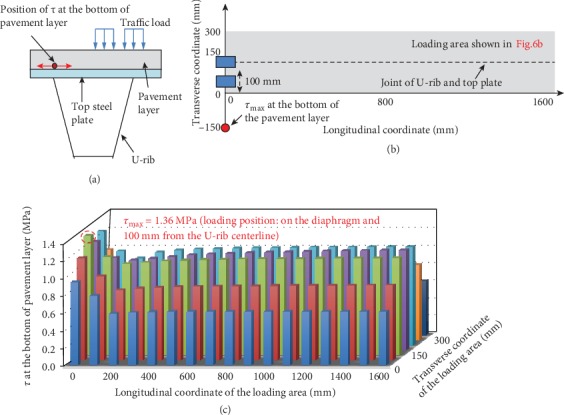
(a) The location of *τ*, (b) the location of *τ*_max_ and the corresponding loading position, and (c) the change of *τ* with the change of loading positions.

Considering the shear resistance between the steel plate and the pavement layer, it is necessary to emphasize the shear stress at the bottom of pavement layer in the transverse direction (*τ*) (Figure 10(a)). The double-wheel load is traversed through the gray area in Figure 10(b), and the relationship between the shear stress (*τ*) and the position of the load is plotted in Figure 10(c).

*τ* reaches the maximum of about 1.36 MPa at the location of near the diaphragm and 150 mm away from the U-rib centerline, when the center point of the double-wheel load is located on the diaphragm and 100 mm away from the U-rib center line. It starts to stabilize at 200 mm from the diaphragm.

### 5.5. Maximum Transverse Tensile Strain at the Top of Pavement Layer (*ε*) and Its Most Unfavorable Loading Position

**Figure 11:**
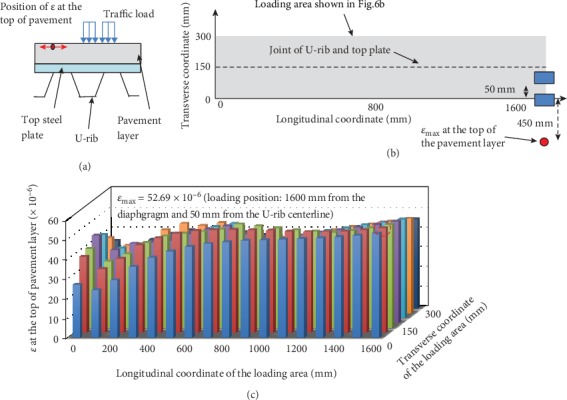
(a) The location of *ε*, (b) the location of *ε*_max_ and the corresponding loading position, and (c) the change of *ε* with the change of loading positions.

To prevent longitudinal fatigue cracking at the top pavement layer, the tensile strain at the top pavement in the transverse direction (*ε*) should be emphasized (Figure 11(a)). The center point of the double-wheel load is traversed through the gray area in Figure 11(b). It is found that when the load is away from the diaphragm, *ε* first decreases and then increases until stabilized.

In particular, when the center point of the double-wheel load is located at mid-span between the two diaphragm plates and 50 mm away from the U-rib centerline, *ε* reaches a maximum of about 52.69 × 10^−6^. The location of the maximum tensile strain occurs near the mid-span and 450 mm from the U-rib centerline.

### 5.6. Maximum Local Deflection of the Top Pavement Layer (*l*_local_) and Its Most Unfavorable Loading Position

**Figure 12:**
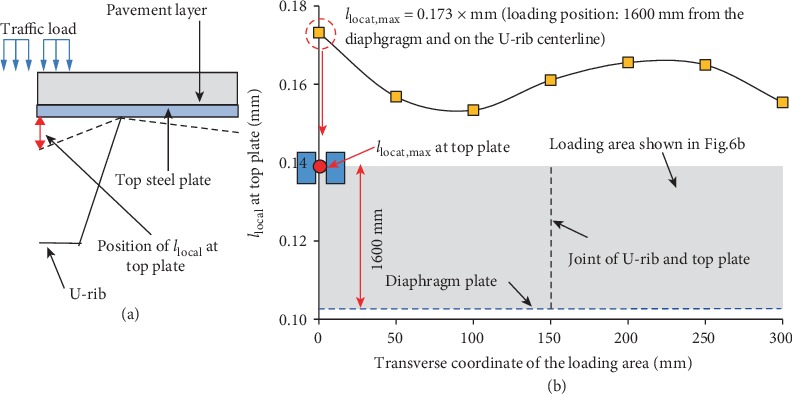
(a) The location of *l*_local_ and (b) the change of *l*_local_ with the change of loading positions.

To prevent too much deflection of the top plate (*l*_local_) and cracking in the pavement, it is necessary to emphasize the local deflection of the top plate (Figure 12(a)). Relevant research [19] shows that the orthotropic steel bridge deck system has significant local effects under the load, and the fatigue cracking failure of the pavement surface can be prevented by controlling the deflection-to-span ratio of the U-rib. The deflection of the pavement layer increases with the load moving away from the diaphragm toward the mid-span. Therefore, the mid-span loading is normally adopted as the critical loading condition, and the load is traversed along the transverse direction only to find the most unfavorable loading position of *l*_local_.

The relationship between the local deflection of the top plate and the transverse position of the load at the mid-span is plotted in Figure 12(b). It is seen that *l*_local_ decreases first and then increases when the load moves from the centerline of the U-rib to the joint between the U-rib and the top plate. When the load moves further toward the mid-span of the two adjacent U-ribs, *l*_local_ increases first and then decreases. In particular, when the center point of the double-wheel load is located at the mid-span and the U-rib centerline, *l*_local_ reaches the maximum of about 0.173 mm.

### 5.7. Summary

According to the calculated results shown in Figures 7–12, the most unfavorable loading positions and the most unfavorable locations of stress, strain, or deflection of each fatigue detail are obtained and summarized in Table 4. The most unfavorable loading positions in Table 4 are then used to calculate the maximum response values of each fatigue detail for samples listed in Table 3. These calculated results will be used to establish the explicit response surface functions between the response values and the structural parameters, which will be detailed in Section 6.

## 6. Establishing Explicit Response Surface Functions

### 6.1. Establishing Response Surface Functions

The response surface methodology (RSM) is based on the experimental or numerical results of the sample group to find the explicit relationships between the response values and the structural parameters. At present, the commonly used response surface functions include elementary functions such as polynomial functions, exponential functions, and logarithmic functions. Given that the multivariable quadratic polynomial has simple expressions and can reflect the coupling relationship between structural parameters, this study will use the quadratic polynomial (as shown in Equation (4)) to characterize the relationship between the fatigue details and the structural parameters. The least squares method is used to determine the fitting parameters of Equation (4) from the data listed in Table 3.

(4) $$\begin{array}{c} y_{j}=f_{j}\left(X\right)=\underset{0\leq m,n\leq 8}{\sum }a_{m,n}x_{m}x_{n}, \end{array}$$

where *y*_*j*_ is the response value of one fatigue detail, such as Δ*σ*_1_, Δ*σ*_2_, Δ*σ*_3_, *τ*, *ε*, and *l*_local_ shown in Table 3. *a*_*m*,*n*_ is the fitting parameter. *x*_*m*_ and *x*_*n*_ are the *m*^th^ and *n*^th^ structural parameters such as *x*_0_, *x*_1_, *x*_2_,…, *x*_8_. Specially, *x*_0_ = 1.

The regressed response surface functions for the six fatigue details are shown in Equations (5)–(10). In particular, for the transverse tensile strain of the top pavement layer (*ε*), the direct use of the quadratic polynomial has a poor fitting result (predicted *R*^2^ only equals to 0.65). Therefore, the tensile strain was converted into tensile stress by multiplying the elastic modulus of the top pavement layer to improve its fitting results as shown in Equation (9). Similarly, since the variation range of the local deflection of the top plate (*l*_local_) is relatively small, the fitting result is not desirable when the multivariate quadratic polynomial is directly used to fit the local deflection of the top plate. Therefore, the inverse of the local deflection-to-span ratio (300/*l*_local_, 300 is the distance between two adjacent ribs) was used for fitting, and the fitting effect can be significantly improved as shown in Equation (10). According to Equations (5)–(10), *R*^2^ of all response surface functions is above 0.93, indicating that the response surface functions described above can accurately predict the response values in the sample space.

(1) The stress amplitude at the welding joint between the top plate and the U-rib in the transverse direction (Δ*σ*_1_, MPa): (predicted *R*^2^ = 0.9925)
  (5) $$\begin{array}{c} \Delta \sigma_{1}=205.21‐5.86x_{1}+1.11x_{2}+0.00746x_{4}-2.497x_{5}-0.00308x_{6}-2.3804x_{7}-0.00409x_{8}+0.0351x_{1}x_{5}+6.626\times 10^{-5}x_{1}x_{6}+0.035x_{1}x_{7}+6.508\times 10^{-5}x_{1}x_{8}-1.326\times 10^{-5}x_{5}x_{6}+0.0191x_{5}x_{7}+2.99\times 10^{-5}x_{5}x_{8}+1.374\times 10^{-5}x_{6}x_{7}+6.681\times 10^{-9}x_{6}x_{8}+0.0272x_{1}x_{1}-0.0346x_{2}x_{2}-1.231\times 10^{-6}x_{3}x_{3}+0.00991x_{5}x_{5}+5.215\times 10^{-8}x_{6}x_{6}+0.00893x_{7}x_{7}+5.464\times 10^{-8}x_{8}x_{8} \end{array}$$
(2) The stress amplitude at the opening of the diaphragm plate in the height direction (Δ*σ*_2_, MPa): (predicted *R*^2^ = 0.9984)
  (6) $$\begin{array}{c} {\Delta \sigma }_{2}=119.29‐0.51x_{1}-0.71x_{2}-6.99x_{3}+0.00756x_{4}-0.338x_{5}-0.000191x_{6}-0.258x_{7}-0.0006234x_{8}+0.00648x_{1}x_{2}+0.00948x_{1}x_{3}+0.0028x_{1}x_{5}+4.056\times 10^{-6}x_{1}x_{8}-5.743\times 10^{-5}x_{2}x_{4}+0.00835x_{2}x_{5}+5.792\times 10^{-6}x_{2}x_{6}+0.00806x_{2}x_{7}+1.463\times 10^{-5}x_{2}x_{8}+6.078\times 10^{-5}x_{3}x_{4}+0.0106x_{3}x_{5}+5.743\times 10^{-6}x_{3}x_{6}+0.00999x_{3}x_{7}+1.696\times 10^{-5}x_{3}x_{8}-2.838\times 10^{-5}x_{4}x_{5}-2.702\times 10^{-5}x_{4}x_{7}-5.262\times 10^{-8}x_{4}x_{8}-4.291\times 10^{-6}x_{5}x_{6}-6.153\times 10^{-6}x_{7}x_{8}-0.00734x_{2}x_{2}+0.127x_{3}x_{3}-7.519\times 10^{-7}x_{4}x_{4}+3.817\times 10^{-9}x_{6}x_{6}+1.029\times 10^{-8}x_{8}x_{8} \end{array}$$
(3) The stress amplitude at the inner side of stiffener in the oblique rib direction (Δ*σ*_3_, MPa): (predicted *R*^2^ = 0.9964)
  (7) Δσ3=109.29+0.45x1−4.99x2−1.218x3+0.00527x4−0.9597x5−0.000763x6−0.891x7−0.00167x8+0.0529x1x2+0.00647x1x5+8.236×10−6x1x6+0.00581x1x7+1.134×10−5x1x8+0.026x2x3+0.0148x2x5+9.776×10−6x2x6+0.0148x2x6+2.43×10−5x2x8+0.00312x3x5+0.00278x3x7+5.611×10−6x3x8−6.191×10−6x5x6+0.00593x5x7+9.67×10−6x5x8+3.122×10−6x6x7−1.97×10−6x7x8−0.0445x1x1+0.0438x2x2+0.0134x3x3−8.688×10−7x4x+40.00313x5x5+1.746×10−8x6x6+0.00287x7x7+2.403×10−8x8x8
(4) The shear stress at the bottom pavement layer in the transverse direction (*τ*, MPa): (predicted *R*^2^ = 0.9880)
  (8) $$\begin{array}{c} \tau =3.44-0.19x_{1}-0.033x_{2}-0.030x_{5}+1.15\times 10^{-4}x_{6}-0.019x_{7}-6.34\times 10^{-7}x_{8}+6.62\times 10^{-4}x_{1}x_{2}+1.39\times 10^{-3}x_{1}x_{5}-8.66\times 10^{-7}x_{1}x_{6}+1.16\times 10^{-3}x_{1}x_{7}+1.65\times 10^{-6}x_{1}x_{8}-1.02\times 10^{-6}x_{2}x_{6}-6.62\times 10^{-7}x_{5}x_{8}-5.61\times 10^{-7}x_{6}x_{7}-1.24\times 10^{-9}x_{6}x_{8}-1.78\times 10^{-7}x_{7}x_{8}+1.51\times 10^{-3}x_{1}x_{1}+8.56\times 10^{-4}x_{2}x_{2}+7.22\times 10^{-5}x_{5}x_{5}-9.63\times 10^{-10}x_{6}x_{6}+4.62\times 10^{-10}x_{8}x_{8} \end{array}$$
(5) The tensile strain at the top pavement layer in the transverse direction (*ε*, ×10^−6^): (predicted *R*^2^ = 0.9306)
  (9) $$\begin{array}{c} \varepsilon x_{8}/10^{6}=2.83-0.12x_{1}-0.09x_{2}-3.37\times 10^{-4}x_{4}-0.03\;x_{5}+4.37\times 10^{-4}x_{6}-0.02\;x_{7}-1.41\times 10^{-4}x_{8}+1.84\times 10^{-3}x_{1}x_{5}-4.28\times 10^{-6}x_{1}x_{6}+1.71\times 10^{-3}x_{1}x_{7}+2.38\times 10^{-6}x_{1}x_{8}+3.63\times 10^{-5}x_{2}x_{4}-3.35\times 10^{-6}x_{5}x_{6}+1.13\times 10^{-6}x_{5}x_{8}-2.41\times 10^{-6}x_{6}x_{7}-6.95\times 10^{-9}x_{6}x_{8}-1.61\times 10^{-9}x_{6}x_{6}+3.74\times 10^{-9}x_{8}x_{8} \end{array}$$
(6) The local deflection of the top plate (*l*_local_, mm): (predicted *R*^2^ = 0.9495)
  (10) $$\begin{array}{c} 300/l_{local}=5344.17+149.68x_{1}+199.5x_{2}-18.73x_{3}-2.55x_{4}-68.94x_{5}-0.117x_{6}-49.15x_{7}-0.291x_{8}-8.315x_{1}x_{3}+0.00646x_{1}x_{6}+0.00972x_{1}x_{8}+0.00657x_{3}x_{6}+0.0066x_{3}x_{8}-0.0364x_{4}x_{5}-0.0363x_{4}x_{7}+0.0075x_{5}x_{6}+3.006x_{5}x_{7}+0.00522x_{5}x_{8}+0.0108x_{7}x_{8}+0.000587x_{4}x_{4}+1.754x_{5}x_{5}-7.922\times 10^{‐6}x_{6}x_{6}+1.718x_{7}x_{7}-7.763\times 10^{‐6}x_{8}x_{8} \end{array}$$

It is seen from the response surface functions that the structural parameters have different degrees of influence on different response values. For Δ*σ*_1_ and *ε*, their response surface functions do not include *x*_3_ (thickness of the diaphragm), indicating that the influence of the thickness of the diaphragm (*x*_3_) on Δ*σ*_1_ and *ε* is much less than that of the other seven structural parameters. For *τ*, the response surface function does not include *x*_3_ (thickness of the diaphragm) and *x*_4_ (spacing of the diaphragm), indicating that they have much less influence than that of the other six structural parameters. For Δ*σ*_2_, Δ*σ*_3_, and *l*_local_, it is found that their response surface functions contain all the structural parameters, indicating that Δ*σ*_2_, Δ*σ*_3_, and *l*_local_ are subject to the influence from all the eight structural parameters.

### 6.2. Correlation of Response Surface Functions

To ensure the applicability of the response surface functions, the correlation between the response surface function and the structural parameters of the initial sample group (shown in Table 3) needs to be tested.

**Figure 13:**
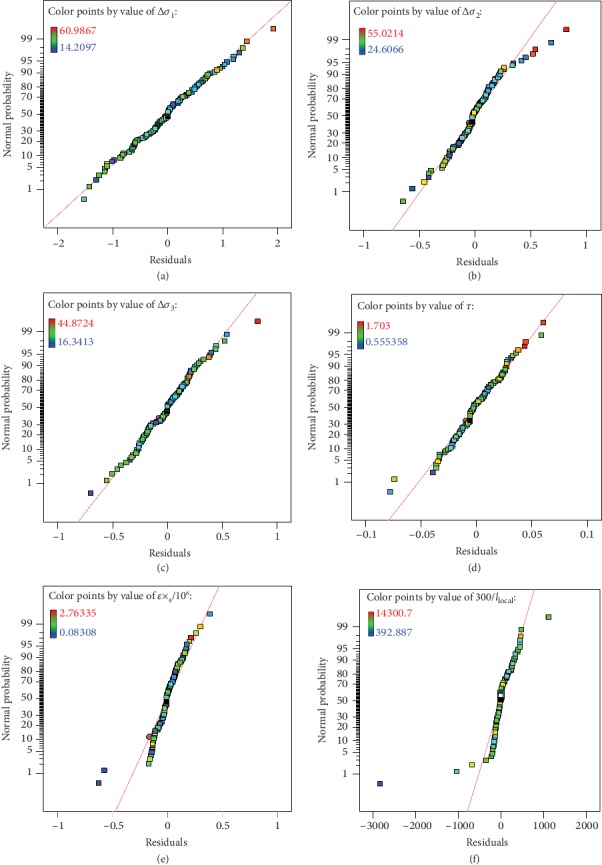
The normal residual plots of six fatigue details.

The normal residual plot is used to show the relationship between the cumulative frequency distribution of the sample results and the cumulative probability distribution of the theoretical normal distribution. If the distribution of each point is approximate to a straight line, the normal distribution assumption of the sample results is acceptable and the response surface functions obtained by RMS are acceptable. The normal residual plots of all six fatigue details are shown as Figure 13. It is seen that the residual points of all fatigues details are distributed in a straight line. This shows that the response surface functions have good applicability to the calculated results of all samples (shown in Table 3).

After the explicit functional relationships between the fatigue details and the structural parameters have been obtained, the optimization can be carried out according to the objectives and constraints, which is detailed in Section 7.

## 7. Nonlinear Optimization Design of Steel Bridge Deck System

The design of the steel bridge deck system can be based on the requirements of both safety and the mass of the system. To balance the safety and the mass, nonlinear optimization is used for design, which is capable of solving the optimization problem with several nonlinear objective functions or constraint functions. The expression of nonlinear optimization is shown in Equation (11). There are some normally used nonlinear optimization algorithms to obtain the optimal result with constraints, including the gradient descent method, Newton method, and conjugate gradient method [38].

(11) $$\begin{array}{c} \left\{\begin{array}{cc} \min F_{\text{obj}}\left(X\right)\\ \text{s}.\text{t}.\;g_{i}\left(X\right)\leq 0 & i=1,2,\cdots ,m\\ \;h_{j}\left(X\right)=0\; & j=1,2,\cdots ,n, \end{array}\right. \end{array}$$

where the *X* in Equation (11) has the same meaning as the *X* in Equation (4), both are the structural parameters to be optimized. *F*_obj_(*X*) is the objective function such as Δ*σ*_1_ and Δ*σ*_2_. *g*_*i*_(*X*) and *h*_*j*_(*X*) are constraint functions such as allowable stress amplitude or deflection. *m* and *n* represent the number of inequality and equality constraint functions, respectively.

Different constraints and optimization objectives will give different optimized results for nonlinear optimization problems. This study will provide both the single-objective optimization and the multiobjective optimization, as detailed follows.

### 7.1. Single-Objective Optimization: Constraints (Structural Safety and Structural Parameter Range) + Single Objective (Structural Mass)

#### 7.1.1. The Constraints (Structural Safety and Structural Parameter Range)

The constraint condition of the single-objective optimization problem mainly considers the safety issue. Moreover, it needs to satisfy the constraints of the structural parameter ranges. According to the relevant provisions “Specifications for Design of Highway Steel Bridge (JTG D64-2015)” [31], the safety constraints of the steel bridge deck system are mainly as follows:

1. *Stress Amplitude*. The stress amplitude of Δ*σ*_1_, Δ*σ*_2_, and Δ*σ*_3_ should not be greater than 30 MPa
2. *Tensile Stress of the Top Pavement Layer in Transverse Direction*. By referring to the “Theory and Method of Pavement Design for Long-Span Bridge Deck” [19], the transverse tensile stress of the top pavement layer should not be greater than 0.7 MPa
3. *Local Deflection-to-Span Ratio*. The local deflection-to-span ratio of the top plate should not be greater than 1/1000, that is, 300/*l*_local_ should not be less than 1000
4. *Shear Stress at the Bottom of the Pavement*. It is required that the bottom of the pavement layer be firmly bonded to the steel plate, and the pull-out test shows that the epoxy asphalt bonding layer has good compatibility with the epoxy zinc-rich anticorrosive coating. The bonding strength is 3.20 MPa at a temperature of 20°C [19]. For the value ranges listed in Table 1, the calculated transverse shear stress at the bottom of the pavement layer are less than 2.1 MPa, which is less than the bonding strength of 3.20 MPa. Therefore, this study does not set any constraints on the shear stress at the bottom of the pavement layer.

#### 7.1.2. The Single Objective (Structural Mass)

The single-objective function mainly considers the mass of the unit area materials. The different single optimization objectives and their objective functions are set as follows.

(1) *Objective 1*: minimize the thickness of the pavement layer, and its objective function is shown in Equation (12):
  (12) $$\begin{array}{c} F_{\text{obj}}=x_{5}+x_{7}, \end{array}$$
  where *x*_5_ is the thickness of the bottom pavement layer and *x*_7_ is the thickness of the top pavement layer
(2) *Objective 2*: minimize the steel used per unit area, and its objective function is shown in Equation (13):
  (13) $$\begin{array}{c} F_{\text{obj}}=m_{\text{steel}}/A_{\text{sys}}, \end{array}$$
  where *m*_steel_ is the mass of the steel materials and *A*_sys_ is the area of the steel bridge deck system
(3) *Objective 3*: minimize the total amount of steel and pavement materials, and its objective function is shown in Equation (14):
  (14) $$\begin{array}{c} F_{\text{obj}}=m_{\text{sys}}/A_{\text{sys}}, \end{array}$$
  where *m*_sys_ is the mass of the steel and the pavement materials. *A*_sys_ is the area of the steel bridge deck system
(4) *Objective 4*: improving the safety of the bridge deck system by strengthening the thickness of the orthotropic steel plate requires *x*_1_ ≥ 14, *x*_2_ ≥ 8, *x*_3_ ≥ 12, and *x*_4_ ≤ 3200. The optimization objective is still to use the minimum total amount of steel and pavement materials per unit area, and its objective function is shown in Equation (14).

#### 7.1.3. The Comparison of the Optimized Results Based on Different Single Objectives

Table 5 shows the comparison of the optimized results for different single-objective optimization made in Section 7.1.2.

For the optimization of Objective 1, the pavement thickness (*x*_5_ and *x*_7_) can be reduced by increasing the thickness of the steel deck plate (*x*_1_), the U-rib (*x*_2_), the diaphragm (*x*_3_), and the elastic modulus (*x*_6_ and *x*_8_) of the pavement material. The thickness of the steel deck plate (*x*_1_) and the elastic modulus of the top pavement layer (*x*_8_) reach the maximum of their value ranges, while the thicknesses of the top and bottom pavement layers (*x*_5_ and *x*_7_) almost reach the minimum of their value ranges to achieve Objective 1.

For the optimization of Objective 2, the thickness of the steel deck plate, the U-rib, and the diaphragm (*x*_1_, *x*_2_, and *x*_3_) can be reduced by increasing the thickness of the pavement layer (*x*_5_ and *x*_7_) and increasing the elastic modulus of the pavement material (*x*_6_ and *x*_8_). The thicknesses and the elastic moduli of the top and bottom pavement layer (*x*_5_, *x*_6_, *x*_7_, and *x*_8_) reach the maximum of their value ranges, while the thickness of the steel deck plate, the U-rib, and the diaphragm (*x*_1_, *x*_2_, and *x*_3_) almost reach the minimum of their value ranges, and the spacing between the two adjacent diaphragms (*x*_4_) reaches the maximum of its value ranges to achieve Objective 2.

For the optimization of Objective 3, since the steel density (about 7.90 t/m^3^) is much higher than that of the asphalt concrete (about 2.45 t/m^3^), the optimization algorithm tends to reduce steel consumption to minimize the total amount of steel and pavement materials. The thickness and the elastic modulus of the top pavement layer (*x*_7_ and *x*_8_) almost reach the maximum of their value ranges, while the thicknesses of the steel deck plate and the U-rib (*x*_1_ and *x*_2_) almost reach the minimum of their value ranges and the spacing between the two adjacent diaphragms (*x*_4_) reaches the maximum of its value ranges to achieve Objective 3. However, the thickness of the diaphragms (*x*_3_) almost reaches the maximum of its value ranges. This shows that the thickness of pavement layer (*x*_5_ and *x*_7_) can be greatly reduced by slightly increasing the thickness of the diaphragms (*x*_3_).The optimization of Objective 4 requires *x*_1_ ≥ 14, *x*_2_ ≥ 8, *x*_3_ ≥ 12, and *x*_4_ ≤ 3200 to improve the safety of the bridge deck system. On the other hand, the optimization principle is similar to Objective 3, that is, by increasing the thicknesses and the elastic moduli of the pavement layer (*x*_5_, *x*_6_, *x*_7_, and *x*_8_) to reduce the consumption of the steel. From the optimized results, the thicknesses of the steel deck plate and the U-rib (*x*_1_ and *x*_2_) almost reach the new minimum value of 14 mm and 8 mm, respectively, as set above, and the spacing between the two adjacent diaphragms (*x*_4_) reaches the new maximum of 3200 mm. Other optimized structural parameters are similar to the optimized results of Objective 3.

### 7.2. Multiobjective Optimization: Constraints (Structural Parameter Ranges) + Objectives (Structural Safety and Structural Mass)

The multiobjective optimization problem only constrains the value range of the structural parameters, while taking the structural safety (values of fatigue details, as calculated from Equations (5)–(10)) and the mass of the unit area materials (*m*_sys_, as calculated from Equation (14)) as the optimization objectives. Moreover, each response value needs to be normalized due to the fact that the units of each response value are different, and the objectives need to be assigned with corresponding weight parameters because of their different importance.

For the safety objectives, different fatigue details have different limits and their values need to be normalized first for combination. The weights for different objective functions and the normalization methods are summarized in Table 6. For the mass objectives, under the fixed loading capacity of the bridge, the lighter the bridge deck system, the larger weight of the vehicle allowed to pass, the more economical the bridge is. Therefore, an objective function is taken as the mass of the unit area materials (*m*_sys_). In this study, the mid-point sample *X* = (16, 10, 15, 3000, 30, 10500, 30, 10500)^*T*^ in the sample space corresponding to *m*_sys_ = 406 kg/m^2^ is used for normalization of the mass of the unit area materials. This result is combined with the normalization of the safety objects by different weights to take both the structural safety and the mass into account.

For the multiobjective optimization composed of the six optimization objects shown in Table 6, five weight combinations are selected for the optimization, as listed in Table 7. In the first group, all weights are the same. In the second group, the weight of the mass of the unit area materials (*w*_6_) is dominant. In the third group, the weights of the overall safety (*w*_1_ ~ *w*_5_) are dominant. In the fourth group, the weights of the orthotropic steel plate safety (*w*_1_ ~ *w*_3_) are dominant. In the fifth group, the weights of the pavement safety (*w*_4_, *w*_5_) are dominant.

Table 7 summarizes the optimized structural parameters from the multiobjective function optimization design. It is seen that the optimized results of the structural parameters vary with the weights of the objectives. By comparing the second group with others, it is seen that when only the mass of the unit area materials is considered, the structural parameters will be relaxed within the allowable range of the fatigue details. Comparing the third, fourth, and fifth groups of Table 7, it is seen that when safety of different parts is considered, the structural parameters of the pavement layer (*x*_5_, *x*_6_, *x*_7_, and *x*_8_) always reach the maximum of the value ranges, and the structural parameters of orthotropic steel plate (*x*_1_, *x*_2_, *x*_3_, and *x*_4_) vary according to the weight combination. Therefore, it is inferred that increasing the thickness of the pavement (*x*_5_ and *x*_7_) or the elastic modulus of the pavement materials (*x*_6_ and *x*_8_) is a way of satisfying both the safety and mass requirements. Pavement materials with higher elastic modulus such as epoxy asphalt mixture can be used for engineering application.

### 7.3. Comparative Analysis of Nonlinear Optimization Algorithms

Current nonlinear optimization algorithms suitable for computer operation include interior-point, sqp, and active-set [39–41]. The sensitivities of different algorithms to the initial values and the iteration efficiency are different, which may cause differences in the final optimized results. Taking the mass of the unit area materials (*m*_sys_, the calculation is shown as Equation (14)) as the single optimization objective, the initial value sensitivity and the iterative efficiency of the above three algorithms are analyzed. The initial iteration values of the structural parameters are taken as *X*_L_ = (12, 6, 10, 2400, 20, 4000, 20, 4000)^*T*^, *X*_M_ = (16, 10, 15, 3000, 30, 10500, 30, 10500)^*T*^, and *X*_U_ = (20, 14, 20, 3600, 40, 17000, 40, 17000)^*T*^, where *X*_L_, *X*_U_, and *X*_M_ are the lower bound, the upper bound, and the medium bound of the value range in Table 1.

The comparison between the number of iterations and the optimized results of the three optimization algorithms is shown in Table 8. Since the density of the pavement material is much less than that of steel, the three algorithms all tend to reduce the thickness of the steel by increasing the elastic modulus of pavement to achieve the objective of reducing the total mass of steel and pavement in the unit area. In addition, given the initial values for the same structural parameters, though the final optimized results obtained by the three algorithms are basically the same, the final results by the sqp and the active-set algorithms are affected by the initial value of the iteration. The interior-point algorithm is more stable than the above two algorithms, indicating that the interior-point algorithm is more suitable for the optimization analysis of the steel bridge deck system.

## 8. Conclusions

This study proposes a response surface methodology- (RSM-) based nonlinear method for optimizing the steel bridge deck system to simplify the design process and reduce the calculation workload. The optimization method proposed is first to generate a sample space, within which the samples can be evenly distributed by using the Box-Behnken design to improve the accuracy of the response surface functions. The FE method is used to analyze the mechanical responses (fatigue details) of the sample groups. The regression analysis based on RSM is then conducted to obtain the explicit relationships between the six fatigue details and the eight design parameters of the steel bridge deck system. Finally, the nonlinear optimization design of the system is performed. Five constraint functions were selected in this study in terms of the limit stress or strain referring to the relevant codes. Considering the mass and the safety of the steel bridge deck system, six objectives with assigned weights are taken into account to obtain the optimized result.

In summary, three conclusions can be drawn from this study:

- From the calculated results by FE analysis, Δ*σ*_2_ (the stress amplitude at the opening of the diaphragm plate in the height direction) is larger than the stress amplitudes occurring at other parts. Therefore, it is necessary to strengthen the thickness of the steel plate at the opening of the diaphragm or optimize the shape of the opening to prevent fatigue cracking
- It is found that the thickness of pavement on the steel deck can be reduced by increasing the thickness of the steel plate or increasing the elastic modulus of the pavement materials. Because the density of steel is much larger than that of the asphalt pavement materials, increasing the thickness or the elastic modulus of the pavement is an effective method if both the safety and the mass of the steel bridge deck system are considered
- The optimized results by different nonlinear optimization algorithms are affected by the initial value of the iteration. The interior-point algorithm is less sensitive to the initial value and can achieve a stable optimization design results of the steel bridge deck system.

## Figures and Tables

**Table 1:** Structural parameters and their value ranges used for optimization design in this study.

	Parameters	Unit	Value range
*x*_1_	The thickness of the top plate	mm	[12, 20]
*x*_2_	The thickness of the U-rib	mm	[6, 14]
*x*_3_	The thickness of the transverse diaphragm plate	mm	[10, 20]
*x*_4_	The spacing of the transverse diaphragm plate	mm	[2400, 3600]
*x*_5_	The thickness of the bottom pavement layer	mm	[20, 40]
*x*_6_	The elastic modulus of the bottom pavement layer	MPa	[4000,17000]
*x*_7_	The thickness of the top pavement layer	mm	[20, 40]
*x*_8_	The elastic modulus of the top pavement layer	MPa	[4000,17000]
Invariant	The width of the U-rib upper opening	mm	300 (fixed value)
Invariant	The height of the U-rib	mm	300 (fixed value)
Invariant	The width of the U-rib lower opening	mm	180 (fixed value)
Invariant	The center distance of U-ribs	mm	600 (fixed value)
Invariant	The height of the transverse diaphragm plate	mm	700 (fixed value)
Invariant	The opening form of the transverse diaphragm plate	—	Refer to Eurocode 3

**Table 2:** Structural parameters of the bridge deck system for some typical long-span steel bridges in China [1, 19–24].

Bridge name	The thickness of the top plate	The transverse diaphragm plate	The stiffener	The pavement
Su-Tong Yangtze River Highway Bridge	≥14 mm	4 m apart; the opening refers to Japanese specification	U-rib (300 × 300 × 8 × 600)	Double-layer epoxy asphalt (55 mm)
Yangluo Bridge	14 mm	8, 10 mm thick; 3.2 m apart	U-rib (300 × 280 × 6 × 600)	Double-layer epoxy asphalt (60 mm)
The Second Nanjing Yangtze River Bridge	14 mm	10 mm thick; 3.75 m apart	U-rib (320 × 280 × 8 × 600)	One-layer epoxy asphalt (50 mm)
Jiangyin Yangtze River Bridge	12 mm	3.2 m apart	U-rib (300 × 280 × 6 × 600)	Double-layer epoxy asphalt (55 mm)
Nansha Bridge	16~18 mm	3.2 m apart	U-rib (300 × 280 × 8 × 600)	Double-layer epoxy asphalt (65 mm)
Haicang Bridge	12 mm	3.0 m apart	U-rib (300 × 280 × 6 × 600)	Double-layer SMA (65 mm)
Hong Kong-Zhuhai-Macao Bridge	≥18 mm	The opening refers to EU specification	U-rib (300 × 300 × 8 × 600)	GMA (lower) + SMA (upper) (68 mm)

The U-rib parameters are upper opening width (mm) × height (mm) × thickness (mm) × center distance (mm).

**Table 3:** The calculated six fatigue details under the most unfavorable loading locations for the 120 samples generated in this study.

No.	**x**_1_ (mm)	**x**_2_ (mm)	**x**_3_ (mm)	**x**_4_ (mm)	**x**_5_ (mm)	**x**_6_ (MPa)	**x**_7_ (mm)	**x**_8_ (MPa)	*∆ * **σ**_1_ (MPa)	*∆ * **σ**_2_ (MPa)	*∆ * **σ**_3_ (MPa)	**τ** (MPa)	**ε** (×10^−6^)	*l*_local_ (mm)
1	16	6	15	3600	20	10500	40	10500	24.96	35.65	32.17	1.14	66.24	0.054
2	16	6	10	3000	20	10500	30	4000	44.04	55.02	44.87	1.20	184.17	0.089
3	16	14	20	3000	30	4000	20	10500	44.11	28.55	23.33	0.76	117.46	0.056
4	16	14	15	3600	20	10500	20	10500	44.29	37.09	24.61	1.19	151.16	0.067
5	16	10	10	3600	30	10500	40	4000	31.22	49.14	29.00	1.01	128.85	0.056
6	12	14	10	3600	30	10500	30	10500	34.29	47.29	18.68	1.19	107.34	0.047
7	12	6	10	2400	30	10500	30	10500	29.88	48.12	33.76	1.33	87.08	0.047
8	16	10	10	3600	40	4000	30	10500	31.03	46.55	27.63	0.67	67.21	0.046
9	20	14	15	3000	30	10500	20	4000	34.12	35.56	24.65	0.87	137.83	0.050
10	20	6	20	2400	30	10500	30	10500	20.41	26.52	29.64	0.95	65.39	0.038
11	16	6	10	3000	30	4000	20	10500	40.91	52.51	41.71	0.83	105.33	0.072
12	16	10	15	3000	20	17000	40	4000	37.10	36.92	29.93	1.32	158.17	0.061
13	16	10	20	3600	40	17000	30	10500	19.47	26.31	20.96	1.17	55.52	0.034
14	12	6	15	3000	30	10500	40	17000	20.78	31.66	26.98	1.16	45.25	0.034
15	20	6	10	3600	30	10500	30	10500	20.18	47.25	34.12	0.94	56.98	0.048
16	16	6	20	3000	40	10500	30	4000	26.99	28.97	31.66	1.11	99.48	0.054
17	16	14	20	3000	20	10500	30	4000	46.70	29.45	24.90	1.08	185.91	0.066
18	16	6	15	2400	40	10500	40	10500	16.86	30.46	26.94	0.94	44.38	0.028
19	20	10	15	2400	40	10500	30	4000	23.31	33.36	25.86	0.85	95.71	0.037
20	16	6	15	2400	20	10500	20	10500	40.66	38.00	39.73	1.33	127.73	0.070
21	12	10	10	3000	30	4000	30	4000	55.28	52.65	33.18	0.94	20.77	0.083
22	12	10	10	3000	40	10500	40	10500	23.53	43.56	21.55	1.01	51.79	0.031
23	12	10	15	3600	20	10500	30	17000	34.38	36.08	24.31	1.47	94.85	0.054
24	16	10	15	3000	40	4000	20	17000	34.47	34.43	27.31	0.72	65.81	0.045
25	16	10	15	3000	20	4000	20	4000	60.99	39.74	38.24	0.75	21.28	0.096
26	20	10	20	3000	40	10500	40	10500	16.42	24.61	20.80	0.77	43.83	0.026
27	20	14	10	2400	30	10500	30	10500	23.34	44.92	21.58	0.83	74.29	0.030
28	20	10	10	3000	30	17000	30	4000	26.25	48.85	29.83	1.12	115.01	0.047
29	16	10	15	3000	40	17000	20	4000	27.82	35.25	26.31	1.35	114.85	0.047
30	12	10	20	3000	40	10500	20	10500	33.19	28.25	22.86	1.25	99.60	0.047
31	12	14	20	2400	30	10500	30	10500	33.46	26.49	16.61	1.20	97.38	0.038
32	12	6	15	3000	20	4000	30	10500	47.63	38.67	38.30	1.19	126.74	0.079
33	20	14	15	3000	30	10500	40	17000	17.50	30.27	17.61	0.79	43.10	0.023
34	16	14	10	3000	40	10500	30	4000	30.08	47.08	21.93	0.98	122.04	0.043
35	16	6	15	3600	40	10500	20	10500	24.96	35.65	32.17	1.13	66.24	0.054
36	16	14	10	3000	30	4000	40	10500	30.66	45.23	21.21	0.68	75.85	0.037
37	12	10	15	2400	40	10500	30	17000	23.72	31.23	20.04	1.07	51.18	0.028
38	16	10	15	3000	20	17000	20	17000	34.93	36.85	28.99	1.52	106.95	0.056
39	16	14	15	2400	30	17000	30	4000	32.27	34.07	21.64	1.32	125.92	0.043
40	16	10	20	3600	20	4000	30	10500	40.41	30.20	28.67	0.88	118.29	0.065
41	16	10	15	3000	30	10500	30	10500	27.63	34.45	25.45	1.04	87.11	0.042
42	16	10	20	2400	20	17000	30	10500	30.97	27.84	25.94	1.38	103.24	0.045
43	16	14	15	3600	30	4000	30	4000	44.87	36.48	24.60	0.68	178.33	0.067
44	16	10	10	2400	30	10500	20	4000	40.98	51.04	33.44	1.19	157.81	0.061
45	20	10	15	2400	20	10500	30	17000	23.53	33.31	25.59	1.01	66.64	0.034
46	16	10	10	2400	20	4000	30	10500	39.44	49.52	31.59	0.88	105.56	0.053
47	16	10	15	3000	30	10500	30	10500	27.63	34.45	25.45	1.04	87.11	0.042
48	16	10	20	3600	30	10500	20	4000	42.04	31.32	30.24	1.20	187.97	0.077
49	12	10	10	3000	20	10500	20	10500	53.19	53.70	33.29	1.62	174.64	0.083
50	16	10	20	2400	40	4000	30	10500	30.63	26.26	24.71	0.67	71.93	0.038
51	16	14	10	3000	20	10500	30	17000	29.99	46.96	21.66	1.16	85.13	0.040
52	20	10	10	3000	30	4000	30	17000	25.63	45.89	27.84	0.64	51.82	0.037
53	20	10	20	3000	30	17000	30	17000	17.72	25.75	21.93	1.08	50.13	0.029
54	20	10	20	3000	20	10500	20	10500	34.40	29.81	29.41	0.96	111.30	0.057
55	20	10	10	3000	40	10500	20	10500	22.77	46.57	27.30	0.88	73.52	0.038
56	12	10	15	2400	20	10500	30	4000	55.88	37.75	32.12	1.52	208.45	0.080
57	12	10	15	2400	30	17000	40	10500	23.79	32.15	20.69	1.36	68.85	0.031
58	16	10	15	3000	30	10500	30	10500	27.63	34.45	25.45	1.04	87.11	0.042
59	16	6	15	2400	30	17000	30	17000	18.28	31.97	28.73	1.33	49.45	0.032
60	16	14	15	2400	20	10500	40	10500	28.04	32.79	19.58	1.02	85.90	0.034
61	20	6	15	3000	30	10500	40	4000	23.58	35.49	33.55	0.90	92.28	0.051
62	12	10	20	3000	30	4000	30	17000	38.69	27.78	23.99	0.95	75.11	0.047
63	12	6	15	3000	30	10500	20	4000	49.73	40.43	40.88	1.63	209.18	0.096
64	20	10	10	3000	20	10500	40	10500	22.77	46.57	27.30	0.88	73.52	0.038
65	20	10	15	2400	30	4000	40	10500	23.56	32.18	25.05	0.58	59.75	0.032
66	16	10	20	2400	30	10500	20	17000	29.54	27.18	24.82	1.14	79.62	0.040
67	20	10	15	2400	30	17000	20	10500	24.45	34.02	26.54	1.16	84.46	0.038
68	12	10	15	3600	30	4000	40	10500	36.06	34.49	23.97	0.85	77.80	0.049
69	20	10	15	3600	20	10500	30	4000	35.52	38.02	31.40	0.85	155.48	0.068
70	16	10	15	3000	20	4000	40	17000	28.43	33.57	25.30	0.90	62.59	0.039
71	12	14	15	3000	30	10500	20	17000	37.38	34.72	18.44	1.32	100.64	0.046
72	16	10	10	3600	20	17000	30	10500	31.33	49.92	29.39	1.36	113.78	0.057
73	20	14	15	3000	20	4000	30	10500	33.10	34.65	23.60	0.65	92.98	0.044
74	12	10	15	3600	40	10500	30	4000	34.89	36.23	24.62	1.24	137.07	0.058
75	16	10	10	2400	30	10500	40	17000	18.92	42.57	22.60	0.94	44.53	0.025
76	12	6	20	3600	30	10500	30	10500	30.34	29.93	30.16	1.35	74.59	0.059
77	16	6	15	3600	30	17000	30	4000	29.75	38.08	35.57	1.50	119.11	0.071
78	16	10	20	2400	30	10500	40	4000	30.70	27.42	25.71	1.02	120.26	0.045
79	16	10	15	3000	30	10500	30	10500	27.63	34.45	25.45	1.04	87.11	0.042
80	16	10	10	2400	40	17000	30	10500	19.08	43.83	23.39	1.15	59.43	0.028
81	16	6	20	3000	30	4000	40	10500	27.18	27.51	30.56	0.75	55.04	0.045
82	16	6	10	3000	30	17000	40	10500	18.06	44.70	31.04	1.24	50.53	0.037
83	16	14	20	3000	40	10500	30	17000	21.56	24.77	16.34	0.89	50.01	0.026
84	16	10	15	3000	30	10500	30	10500	27.63	34.45	25.45	1.04	87.11	0.042
85	16	10	15	3000	30	10500	30	10500	27.63	34.45	25.45	1.04	87.11	0.042
86	12	10	20	3000	30	17000	30	4000	38.52	29.76	25.53	1.70	162.55	0.062
87	12	14	15	3000	20	17000	30	10500	38.85	35.55	19.33	1.59	131.46	0.052
88	16	14	10	3000	30	17000	20	10500	31.10	48.01	22.56	1.32	108.93	0.044
89	16	6	10	3000	40	10500	30	17000	17.63	43.08	29.81	0.98	34.05	0.032
90	20	10	15	3600	30	17000	40	10500	16.99	32.05	22.64	1.01	51.86	0.032
91	12	10	15	3600	30	17000	20	10500	35.63	37.08	25.40	1.69	127.24	0.061
92	16	10	15	3000	40	17000	40	17000	14.21	29.08	19.14	1.01	30.04	0.021
93	20	10	20	3000	30	4000	30	4000	34.14	29.33	29.23	0.56	131.64	0.763
94	16	14	20	3000	30	17000	40	10500	21.78	25.49	16.82	1.10	68.05	0.029
95	12	10	20	3000	20	10500	40	10500	33.19	28.25	22.86	1.27	99.60	0.047
96	16	6	20	3000	20	10500	30	17000	26.73	28.82	31.37	1.31	68.29	0.050
97	12	10	15	2400	30	4000	20	10500	52.37	36.42	29.63	1.03	129.93	0.066
98	20	14	20	3600	30	10500	30	10500	23.93	26.90	19.47	0.84	79.88	0.037
99	16	6	15	3600	30	4000	30	17000	28.80	34.95	32.98	0.83	43.32	0.051
100	16	14	15	3600	40	10500	40	10500	20.76	30.81	16.66	0.83	51.39	0.028
101	16	14	15	2400	40	10500	20	10500	28.04	32.79	19.58	1.00	85.90	0.034
102	16	10	15	3000	30	10500	30	10500	27.63	34.45	25.45	1.04	87.11	0.042
103	20	6	15	3000	30	10500	20	17000	22.74	35.01	32.79	1.02	58.03	0.046
104	16	14	15	3600	30	17000	30	17000	22.02	32.19	17.59	1.16	62.38	0.032
105	16	6	20	3000	30	17000	20	10500	28.04	29.71	32.69	1.52	93.19	0.057
106	16	14	15	2400	30	4000	30	17000	32.04	32.36	20.34	0.75	65.52	0.035
107	20	10	15	3600	30	4000	20	10500	33.64	36.67	29.70	0.61	94.01	0.057
108	12	14	15	3000	40	4000	30	10500	39.83	33.46	18.47	0.78	90.83	0.045
109	12	10	10	3000	30	17000	30	17000	24.79	45.71	23.01	1.42	63.42	0.036
110	20	6	15	3000	40	4000	30	10500	23.09	33.63	32.08	0.58	49.86	0.042
111	16	6	15	2400	30	4000	30	4000	41.02	37.29	39.27	0.76	151.74	0.069
112	16	10	10	3600	30	10500	20	17000	29.82	48.49	28.07	1.14	83.19	0.050
113	12	6	15	3000	40	17000	30	10500	20.89	32.82	27.94	1.45	52.81	0.038
114	16	10	15	3000	40	4000	40	4000	33.59	34.85	27.55	0.63	113.24	0.050
115	20	6	15	3000	20	17000	30	10500	24.01	36.08	34.14	1.23	80.28	0.052
116	20	14	15	3000	40	17000	30	10500	17.68	31.08	18.11	0.96	57.93	0.026
117	12	14	15	3000	30	10500	40	4000	39.20	35.02	19.12	1.20	152.03	0.053
118	16	10	15	3000	30	10500	30	10500	27.63	34.45	25.45	1.04	87.11	0.042
119	20	10	15	3600	40	10500	30	17000	16.65	30.98	21.91	0.81	36.04	0.028
120	16	10	20	3600	30	10500	40	17000	19.29	25.46	20.38	0.95	39.24	0.030

**Table 4:** The most unfavorable loading locations and the most unfavorable stress or deflection locations for the six fatigue details.

Fatigue details	The most unfavorable stress or deflection locations		The most unfavorable loading locations	
	Longitudinal distance from the transverse diaphragm plate	Transverse distance from the centerline of the U-rib	Longitudinal distance from the transverse diaphragm plate	Transverse distance from the centerline of the U-rib
Δ*σ*_1_	About 0.09 times of diaphragm plates spacing	0.5 times the upper opening width of the U-rib	About 0.09 times of diaphragm plates spacing (tensile) About 0.25 times of diaphragm plates spacing (compressive)	0.5 times the upper opening width of the U-rib (tensile) 0.33 times the upper opening width of the U-rib (compressive)
Δ*σ*_2_	Near the transverse diaphragm plate	—	About 0.09 times of diaphragm plates spacing	0.33 times the upper opening width of the U-rib
Δ*σ*_3_	Near the transverse diaphragm plate	0.5 times the upper opening width of the U-rib	Near the transverse diaphragm plate	At the centerline of the U-rib
*τ*	Near the transverse diaphragm plate	0.5 times the upper opening width of the U-rib	Near the transverse diaphragm plate	0.33 times the upper opening width of the U-rib
*ε*	Mid-span	1.5 times the upper opening width of the U-rib	Mid-span	0.17 times the upper opening width of the U-rib
*l*_local_	Mid-span	At the centerline of the U-rib	Mid-span	At the centerline of the U-rib

**Table 5:** Comparison of optimized structural parameters based on different optimization objectives for the single-objective optimization.

Objective	*x*_1_(mm)	*x*_2_(mm)	*x*_3_(mm)	*x*_4_(mm)	*x*_5_(mm)	*x*_6_(MPa)	*x*_7_(mm)	*x*_8_(MPa)
1: minimize pavement thickness	20	8	20	3471	20	8066	22	17000
2: minimize the steel mass per unit area	12	6	14	3600	40	17000	40	17000
3: minimize the steel and pavement mass per unit area	12	6	19	3600	20	8479	35	17000
4: minimize the steel and pavement mass per unit area (enhance the constraints by *x*_1_ ≥ 14 mm, *x*_2_ ≥ 8 mm, *x*_3_ ≥ 12 mm, *x*_4_ ≤ 3200 mm, and the others are the same as the constraints list below)	14	8	18	3200	20	7579	34	17000

Constraints: Δ*σ*_1_, Δ*σ*_2_, Δ*σ*_3_ < 30*MPa*, *x*_8_*ε* < 0.7*MPa*, *l*_*local*_ < 0.3*mm*, and the value ranges shown in Table 1.

**Table 6:** Weight and normalization of different optimization functions for the multiobjective optimization.

Optimization function	Δ*σ*_1_	Δ*σ*_2_	Δ*σ*_3_	*ε*	*l*_local_	*m*_sys_
Weight	*w*_1_	*w*_2_	*w*_3_	*w*_4_	*w*_5_	*w*_6_
Normalization	Δ*σ*_1_/30	Δ*σ*_2_/30	Δ*σ*_3_/30	*ε*/(175 × 10^−6^)	*l*_local_/0.3	*m*_sys_/406

**Table 7:** Comparison of optimized structural parameters under different weights.

No.	Weight (Δ*σ*_1_ : Δ*σ*_2_ : Δ*σ*_3_ : *ε* : *l*_local_ : *m*_sys_)	*x*_1_	*x*_2_	*x*_3_	*x*_4_	*x*_5_	*x*_6_	*x*_7_	*x*_8_
1	(1 : 1 : 1 : 1 : 1 : 1)	12	6	20	3600	40	17000	40	17000
2	(1 : 1 : 1 : 1 : 1 : 10)	12	6	19	3600	20	13572	35	17000
3	(10 : 10 : 10 : 10 : 10 : 1)	12	14	20	2400	40	17000	40	10032
4	(10 : 10 : 10:1 : 1 : 1)	12	14	20	2400	40	17000	40	17000
5	(1:1:1:1 : 10 : 10:1)	20	6	20	3600	40	17000	40	17000

**Table 8:** Comparison of optimized results by different algorithms.

Algorithm	Initial value	Iteration times	Optimized parameter combination	Optimized results (kg/m^2^)
Interior-point	*X*_L_	97	(12,6,18,3200,20,8134,36,17000)^*T*^	327.1
Interior-point	*X*_M_	80	(12,6,18,3200,20,8134,36,17000)^*T*^	327.1
Interior-point	*X*_R_	48	(12,6,18,3200,20,8134,36,17000)^*T*^	327.1
sqp	*X*_L_	46	(12,6,18,3200,20,8134,36,17000)^*T*^	327.1
sqp	*X*_M_	39	(12,6,18,3200,20,8134,36,17000)^*T*^	327.1
sqp	*X*_R_	8	(12,6,16,3200,40,17000,30,17000)^*T*^	355.5
Active-set	*X*_L_	34	(12,6,18,3200,20,8134,36,17000)^*T*^	327.1
Active-set	*X*_M_	88	(12,6,18,3200,34,8366,29,12880)^*T*^	344.7
Active-set	*X*_R_	9	(12,6,16,3200,40,17000,30,17000)^*T*^	355.5
